# The timing of tuberculosis after isoniazid preventive therapy among gold miners in South Africa: a prospective cohort study

**DOI:** 10.1186/s12916-016-0589-3

**Published:** 2016-03-23

**Authors:** Sabine M. Hermans, Alison D. Grant, Violet Chihota, James J. Lewis, Emilia Vynnycky, Gavin J. Churchyard, Katherine L. Fielding

**Affiliations:** TB Centre, London School of Hygiene & Tropical Medicine, London, UK; Department of Global Health, Academic Medical Center, University of Amsterdam, Amsterdam Institute for Global Health and Development, Amsterdam, The Netherlands; Desmond Tutu HIV Centre, Institute for Infectious Diseases and Molecular Medicine, University of Cape Town, Cape Town, South Africa; Department of Internal Medicine, School of Medicine, Makerere University College of Health Sciences, Kampala, Uganda; The School of Public Health, University of the Witwatersrand, Johannesburg, South Africa; School of Nursing & Public Health (Africa Centre for Population Health), University of KwaZulu-Natal, Durban, South Africa; Aurum Institute, Johannesburg, South Africa; Public Health England, London, UK; Advancing Care and Treatment for TB and HIV, MRC Collaborating Centre of Excellence, Johannesburg, South Africa

**Keywords:** Isoniazid preventive therapy, Latent infection, Reactivation, Reinfection, Tuberculosis

## Abstract

**Background:**

The durability of isoniazid preventive therapy (IPT) in preventing tuberculosis (TB) is limited in high-prevalence settings. The underlying mechanism (reactivation of persistent latent TB or reinfection) is not known. We aimed to investigate the timing of TB incidence during and after IPT and associated risk factors in a very high TB and HIV-prevalence setting, and to compare the observed rate with a modelled estimate of TB incidence rate after IPT due to reinfection.

**Methods:**

In a post-hoc analysis of a cluster-randomized trial of community-wide IPT among South African gold miners, all intervention arm participants that were dispensed IPT for at least one of the intended 9 months were included. An incident TB case was defined as any participant with a positive sputum smear or culture, or with a clinical TB diagnosis assigned by a senior study clinician. Crude TB incidence rates were calculated during and after IPT, overall and by follow-up time. HIV status was not available. Multivariable Cox regression was used to analyse risk factors by follow-up time after IPT. Estimates from a published mathematical model of trial data were used to calculate the average reinfection TB incidence in the first year after IPT.

**Results:**

Among 18,520 participants (96 % male, mean age 41 years, median follow-up 2.1 years), 708 developed TB. The TB incidence rate during the intended IPT period was 1.3/100 person-years (pyrs; 95 % confidence interval (CI), 1.0–1.6) and afterwards 2.3/100 pyrs (95 % CI, 1.9–2.7). TB incidence increased within 6 months followed by a stable rate over time. There was no evidence for changing risk factors for TB disease over time after miners stopped IPT. The average TB incidence rate attributable to reinfection in the first year was estimated at 1.3/100 pyrs, compared to an observed rate of 2.2/100 pyrs (95 % CI, 1.8–2.7).

**Conclusions:**

The durability of protection by IPT was lost within 6–12 months in this setting with a high HIV prevalence and a high annual risk of *M. tuberculosis* infection. The observed rate was higher than the modelled rate, suggesting that reactivation of persistent latent infection played a role in the rapid return to baseline TB incidence.

**Electronic supplementary material:**

The online version of this article (doi:10.1186/s12916-016-0589-3) contains supplementary material, which is available to authorized users.

## Background

The world has seen an upsurge in tuberculosis (TB) incidence since the advent of the human immunodeficiency virus (HIV) epidemic in the 1990s. This rise was mainly seen in the sub-Saharan African region, which accounts for 74 % of the annual 1.2 million people living with HIV who develop TB [1]. In southern Africa, HIV prevalence among people with TB disease is around 50 %.

Isoniazid preventive therapy (IPT) is part of the World Health Organization (WHO)’s 3Is strategy formulated to reduce the burden of TB in people living with HIV [2]. A 6–12 month course of daily isoniazid has been shown to reduce TB incidence in those with a positive tuberculin skin test (TST) by an average of 60 % [3]. In a recent randomised controlled trial of IPT in patients on antiretroviral therapy (ART), those with a negative TST at enrolment also benefitted [4], although these participants could have undergone TST conversion whilst on ART [5]. A large cluster-randomised trial of community-wide IPT among 78,744 gold mine workers in South Africa, the Thibela TB study [6], did not find a protective effect of IPT on TB incidence or TB prevalence at a population level. At an individual level, the direct protective effect of IPT was calculated in the subset of the study population included in the baseline prevalence survey, which was a random sample of the total workforce. This showed a 58 % (95 % confidence interval (CI), 12–80 %) lower incidence rate of TB during the 9 months on IPT versus that in a control cohort. However, this protective effect disappeared within the first 9 months after the intended IPT period.

Other studies have also shown that the effect of IPT wanes over time in settings with high annual risks of *Mycobacterium tuberculosis* infection [4, 7–9]. Few IPT trials have reported data on long-term TB incidence rates after IPT discontinuation, and on how these rates change over time. Additional file 1: Table S1 gives an overview of long-term follow-up data of the adult trials. The Bethel studies in the pre-HIV era showed a durable effect over a long period of time, but were performed in a context of reducing TB transmission and other TB control strategies [10, 11]. In contrast, the studies performed in TB high-prevalence settings showed a rapid increase in TB incidence rates after IPT cessation. The underlying mechanism (reactivation of persistent latent infection or reinfection) is not well understood.

The relatively small numbers of events in these cohorts have limited the power to investigate the timing of TB occurring after IPT cessation and the change of associated risk factors over time. We therefore set out to analyse TB incidence in the entire Thibela TB study population that had received IPT. This population was almost four times as large as the IPT cohort used to calculate the direct effect of the intervention, which were the participants included in the baseline survey only (a random sample of 1,000 miners per cluster on whom more in-depth data was collected) [6].

**Fig. 1:**
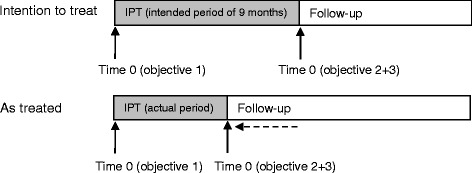
Start of the risk period (time 0) in the intention-to-treat and as-treated analyses. Follow-up was maximum 12 months after the end of intended isoniazid preventive therapy for the last participant in each cluster.

Our study objectives were four-fold: to estimate the TB incidence rate during and after IPT (objective 1); to investigate risk factors associated with incident TB after IPT (objective 2); and to determine whether these differed over time after the end of IPT (effect modification by follow-up period, objective 3). All three analyses used both the intended and actual duration of IPT (Fig. 1). Our final objective was to compare the observed incidence of TB in the first year after IPT with a crude estimate of the average incidence rate of TB disease due to reinfection that might have occurred during that period (objective 4).

## Methods

### Study design and setting: Thibela TB trial

This was a post-hoc analysis of data from the Thibela TB study, a cluster-randomised trial to investigate the impact of community-wide IPT on TB incidence and prevalence compared to standard of care among 15 clusters (mine shafts) of gold mine workers in South Africa [6]. Clusters were randomised to the intervention (TB screening offered to the entire cluster linked to treatment for TB disease or infection, as appropriate; eight clusters) or control (standard of care, seven clusters) arm [12]. The study methods are described in full elsewhere [12].

This analysis focused solely on the eight intervention clusters. Within each cluster, the same procedure was followed for enrolment and follow-up (Additional file 1: Figure S1). All mine workers within the cluster were invited to participate [13]. As 89 % of the workforce was estimated to be infected with *M. tuberculosis*, TST was deemed unnecessary [14]. Consenting miners were screened for TB using a symptom screen and a chest X-ray; a sputum smear and culture was ordered for miners with signs or symptoms suggestive of TB. Positive laboratory results and/or high clinical suspicion were reason for referral to the mine health services for further investigation and, if necessary, TB treatment. Participants without evidence of active TB or contra-indications to IPT were offered 9 months of IPT (300 mg isoniazid daily) [15]. During IPT, participants were seen monthly by study staff for dispensing of study medication, and a symptom screen for TB and adverse events. Symptomatic patients were referred to the mine health services for further work-up and management. After cessation of IPT, participants were evaluated for incident TB during 6-monthly or annual routine chest X-ray screening by occupational health services or after self-presentation with symptoms to the mine health services.

### Population included in post-hoc analysis

All participants in the eight intervention clusters who were prescribed IPT at least once and who were permanent employees were included in this analysis. For analyses involving follow-up after the end of IPT, only participants who had completed the IPT period without a TB diagnosis, alive and still in the workforce, were included. In each cluster, follow-up was continued until 1 year after the end of the last participant’s intended IPT period.

### Definitions and measurement of variables

Baseline characteristics of study participants were collected by a questionnaire at enrolment into the intervention and prior to initiation of IPT. At the request of the trade unions, no HIV testing was performed for study purposes. However, use of ART as concomitant medication was asked with respect to assessing possible adverse events. HIV prevalence among miners was estimated at 29 % in 2000 [16]. IPT dispensing dates were recorded; the number of monthly refill visits was used as a proxy of adherence (≥6 defined as optimal, 6 months being a recommended duration for IPT at the time the trial was designed [17]). The end of the intended IPT period was defined as 270 days from the first prescription date and the end of the actual IPT period as 30 days from the last dispensing date.

For estimation of the TB incidence rate, the start of the risk period was defined as the date of IPT initiation (objective 1); for the risk factor analyses the risk period started at the end of the intended or actual IPT period (objectives 2 and 3).

Miners with symptoms or signs suggesting TB were investigated and treated by the mine health services. Data on these episodes were obtained from record review. Routine diagnostic workup included sputum smear microscopy and a chest X-ray. Only one mining company routinely used mycobacterial culture to evaluate all miners with signs/symptoms suggestive of TB, the others only for those with a history of prior TB treatment. However, during the study, we endeavoured, following additional consent, to collect an additional sputum sample for smear microscopy and culture, though full coverage of this was not achieved. Cultures showing only non-tuberculous mycobacteria were not considered as TB. TB diagnoses were categorised as definite (2 positive smears or 1 positive culture for *M. tuberculosis*, or histological evidence of TB at autopsy in TB cases only ascertained post-mortem), probable (1 positive smear or culture with unidentified mycobacteria) or possible (clinical or radiological signs and symptoms, but no or negative smear/culture results). Medical records of participants with possible TB were reviewed by senior study clinicians, masked to study arm, to arbitrate on whether to include as incident TB.

Employment records were used to determine dates of employment and reasons for leaving the workforce (including deaths). Miners who died and underwent autopsy which revealed histological evidence of TB were included as incident TB cases.

The end of the risk period was defined as the earliest of TB treatment initiation (irrespective of the method of diagnosis), termination from the workforce, death or the end of follow-up. Participants who left the workforce due to being ‘medically boarded’ (discontinuing employment for a medical reason) were all evaluated by the mine healthcare system for active TB prior to the end of employment, and we therefore assumed that none of these represented missed TB diagnoses.

### Statistical methods

The primary analyses were carried out using the intended IPT period to define the end of IPT (intention-to-treat (ITT), Fig. 1). Three sensitivity analyses were conducted: an as-treated (AT) analysis using the actual end date of IPT (Fig. 1), an AT analysis restricted to those with optimal adherence (defined as having been dispensed ≥6 months of IPT, see below), and a repeat analysis of both the ITT and AT analyses with a stricter TB case definition restricted to definite and probable TB diagnoses.

We calculated the overall TB incidence rate after initiation of IPT and period-specific rates during and after IPT. Poisson regression analyses adjusted for clustering by mine shaft were used to calculate TB incidence rates and to determine the rate ratio for TB after stopping IPT compared to during IPT. We investigated trends in TB incidence rates over time after the end of IPT using tests for linearity and departure from linearity.

Multivariable Cox regression analysis was used to investigate risk factors for TB following the end of IPT. We included factors we considered a priori as risk factors for TB (sex, age, previous TB, number of years in the workforce, type of work (reduced ventilation with underground work), housing (crowding in hostels), self-reported use of ART and number of times IPT was dispensed). The potential confounder (country of origin) was retained in the model if inclusion led to a change in hazard ratio estimate. In case of co-linearity, the more important of the co-linear variables was determined by literature review and retained. We considered previous TB might be on the causal pathway as it has been shown to be a risk factor for recurrent TB [18, 19]. We therefore examined the effect of dropping this variable in the final multivariable Cox model. The effect of excluding self-reported ART use was also examined. The final model was adjusted for calendar time.

Clustering was controlled for with a fixed effect for cluster in all regression analyses. Random effects analysis was not used due to small number of clusters [20]. Cluster estimates were not reported.

Departure from the proportional hazards assumption was tested in objective 3: we incorporated interaction terms between the time period of follow-up and the a priori risk factors. To maximise power, we used unadjusted analyses and two time periods: <12 and ≥12 months after stopping IPT. This time split was chosen to correspond with the expected timing of different underlying mechanisms (reactivation of inadequately treated latent infection <12 months versus reinfection ≥12 months), extrapolated from a study of recurrent disease after treatment [21].

For the AT analysis, these procedures were repeated using the actual end date of IPT as defined above. Two-sided *P* values in regression analyses were derived from likelihood ratio tests. Departure from linearity was tested for all ordered categorical variables. The analyses were conducted using STATA version SE 12.1 (College Station, Texas, USA).

### Comparison between observed and modelled TB incidence in the first year after IPT

We applied base-case assumptions used in the published mathematical model of the Thibela study data to calculate a crude estimate of the average incidence rate of TB disease attributable to reinfection in the first year after IPT [22]. We did this overall and by cluster to allow for the varying annual risk of infection per cluster. We thereby assumed an annual risk of infection (averaged across clusters) before the intervention of 20 % per year and, based on outputs from the original model [22], a reduction in the annual risk of infection ranging between 11–20 % after IPT was introduced. For all clusters we assumed an HIV prevalence of 30 % of whom 25 % had a CD4 count below 200 cells/μL (and all of whom were eligible for and on ART), an initial pre-treatment loss to follow-up of 40 % and an average time to detection of 1 year. The age distribution and prevalence of silicosis depended on the cluster and were based on data collected as part of the study. The origin of these assumptions are explained in detail elsewhere [22]. Furthermore, also based on the original model, we assumed that reinfection, but no progression, could take place during IPT. We then calculated the observed TB incidence rate in the first 12 months after IPT (ITT), overall and per cluster, and compared these rates to the modelled estimates of TB incidence.

### Ethical review

The Research Ethics Committees of University of KwaZulu-Natal, South Africa, and the London School of Hygiene & Tropical Medicine (LSHTM) as well as the South African Medicines Control Council and the South African Safety in Mines Research Advisory Committee gave approval for the Thibela TB study. The LSHTM ethics committee gave approval for this post-hoc secondary data analysis. Miners who participated in the intervention provided written or witnessed oral informed consent.

## Results

Between July 2006 and February 2010, 18,520 participants were prescribed IPT at least once and were permanent employees. Their baseline characteristics are described in Table 1; 96 % were men, in keeping with the demographics of this workforce, with a median age of 41 years and a median time in the mining workforce of 18 years. Overall, 57 % of mine workers were South African, 91 % worked mainly underground, 59 % lived in a hostel, and 12 % reported having had TB previously and 2.7 % as being on ART. IPT was dispensed six or more times to 11,293 (61 %) participants, three to five times to 2,125 (12 %), and once or twice to 5,102 (28 %) participants.

**Table 1:** Baseline characteristics of the study population (n = 18,520)

Characteristic		Total, n (col %) ^a^
Sex	Male	17,763 (95.9)
Age, years	≤29	2,456 (13.3)
	30–39	4,919 (26.6)
	40–49	7,782 (42.0)
	≥50	3,362 (18.2)
Country of origin	South Africa	10,501 (56.7)
	Lesotho	5,178 (28.0)
	Mozambique	1,854 (10.0)
	Other	979 (5.3)
Years in workforce	0–9	4,874 (26.4)
	10–19	5,017 (27.2)
	20–29	5,846 (31.7)
	≥30	2,724 (14.8)
Type of work	Underground	16,821 (91.2)
Type of housing	Hostel	10,913 (58.9)
Previous TB ^b^		2,212 (12.0)
Previous IPT		79 (0.4)
Self-reported ART use		521 (2.8)

^a^ Missing data (n): sex (1), age (46), country of origin (8), years in workforce (59), type of work (77), type of residence (1), previous TB (14), previous IPT (38), self-reported ART use (20)

^b^ Timing of previous TB episodes: median 4.0 years (IQR, 1.8–8.9 years), 5 had missing TB dates; 265 (12 %) episodes occurred in year prior to IPT

ART, Antiretroviral therapy; IPT, Isoniazid preventive therapy; IQR, Interquartile range; N, Number; TB, Tuberculosis

### TB incidence rate during and after IPT

**Fig. 2:**
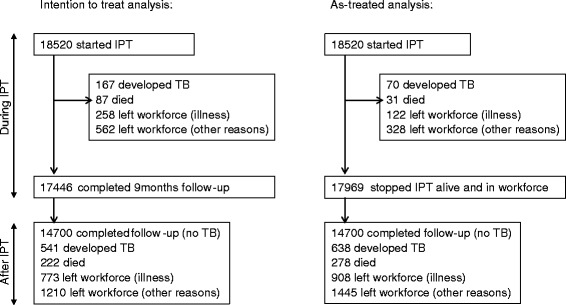
Participant flow and outcomes in the intention-to-treat and as-treated analyses. The duration on isoniazid preventive therapy was defined as the intended duration (intention-to-treat analysis) and 30 days from the last dispensing date (as-treated analysis)

Across all clusters, 708 participants developed TB during 37,321 person-years (pyrs). Of these, 55 % were definite, 12 % probable and 33 % possible diagnoses; 541 were diagnosed after the intended end of IPT (ITT analysis) and 638 after the actual end of IPT (AT analysis, Fig. 2). The median follow-up time, including the IPT period, was 2.1 years and varied by cluster (ranging between 1.9 and 2.5 years).

In the ITT analysis, the overall TB incidence rate on IPT was 1.3/100 pyrs (95 % CI, 1.0–1.6), whereas after IPT it was 2.3 (95 % CI, 1.9–2.7) with a rate ratio (RR) of 1.8 (95 % CI, 1.4–2.3). In the AT analysis, the rate on IPT was 0.7/100 pyrs (95 % CI, 0.5–1.0), whereas after IPT it was 2.3/100 pyrs (95 % CI, 1.9–2.7) with a RR of 3.1 (95 % CI, 2.3–4.3). Among those in the AT analysis who took 6 months or more of IPT, the incidence rate on IPT was 0.3/100 pyrs (95 % CI, 0.2–0.4) and off IPT 2.2/100 pyrs (95 % CI, 1.8–2.7), with a RR of 6.7 (95 % CI, 5.6–8.0).

**Fig. 3:**
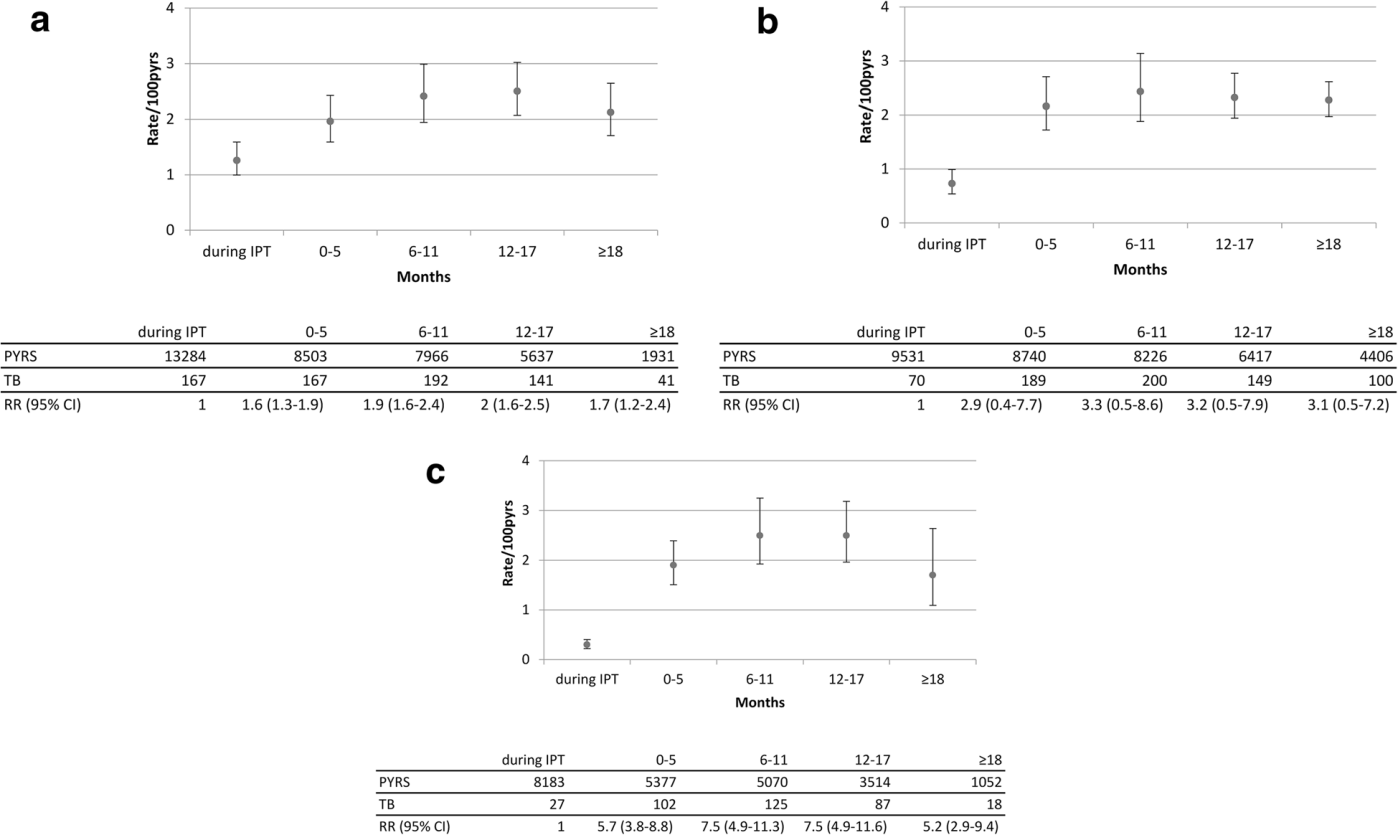
Rates of TB incidence over time during and after IPT in the Thibela TB study. (**a**) intention-to-treat analysis (intended duration of IPT), (**b**) as-treated analysis (actual duration of IPT) and (**c**) as-treated analysis among the subset of optimal adherers (>6 months of IPT)). Rates were adjusted for clustering by cluster. *P* values for overall association between TB incidence and time period of follow-up: (a) <0.001, (b) <0.001 and (c) <0.001. *P* values for tests for linearity in TB incidence rates over time since IPT cessation: (a) 0.043, (b) 0.633 and (c) 0.077. *P* values for tests for departure from linearity in TB incidence rates over time since IPT cessation: (a) 0.071, (b) 0.533 and (c) 0.003. CI, Confidence interval; IPT, Isoniazid preventive therapy; pyrs, Person-years

After the end of IPT, from the test for linearity, there was some evidence for an increasing rate over time in the ITT analysis and in the subgroup who took 6 months or more of IPT, but not in the AT analysis (Fig. 3). The results of the sensitivity analyses excluding possible TB diagnoses were similar (Additional file 1: Figure S2). As expected, the overall TB incidence rates were lower, but the overall pattern of changes of TB incidence over time was similar.

### Risk factors associated with incident TB after IPT

A total of 17,446 (94 %) participants completed the intended IPT period of 9 months alive, in the workforce and not taking TB treatment, and formed the cohort for the risk factor analysis for incident TB after IPT (Fig. 2). They contributed 24,037 person-years, a median follow-up of 1.4 years. Neither their baseline characteristics nor the proportion of participants who took 6 months or more of IPT differed from the original cohort (Additional file 1: Table S2). Characteristics of participants leaving the workforce for reasons other than ill health were compared to those of all participants. On average, these participants were older, had been in the workforce longer and had had TB previously (Additional file 1: Table S3).

After adjusting for sex, country of origin, self-reported ART use and duration of IPT taken, there was strong evidence of an increased hazard of TB following IPT among participants who were older, doing underground rather than surface work or had had TB before (Table 2). A decreased hazard of TB was found in those not living in a hostel or who had more IPT dispensed. Age and years in the workforce were found to be co-linear; we retained age in the model as this had been previously shown to be the stronger risk factor for TB [23]. Excluding previous TB or self-reported ART use from the model did not change the results, nor did adjustment for calendar time.

**Table 2:** Risk factors for TB incidence after IPT (intention-to-treat)

Total		n (%)	pyrs	TB	HR (95 % CI) ^a^	*P* value	aHR (95 % CI) ^a,f^	*P* value
Sex	Male	16,704 (95.8)	23,036	523	1	0.08	1	0.73
	Female	741 (4.2)	999	18	0.67 (0.42–1.08)		1.09 (0.67–1.80)	
Age, years ^b^	≤29	2,370 (13.6)	3,221	30	1	<0.001	1	<0.001
	30–39	4,677 (26.8)	6,660	131	2.13 (1.43–3.17)		2.18 (1.46–3.25)	
	40–49	7,363 (42.2)	10,291	276	2.81 (1.92–4.10)		2.84 (1.92–4.18)	
	≥50	3,035 (17.4)	3,863	104	2.69 (1.79–4.06)		2.77 (1.81–4.23)	
Country of origin	South Africa	10,008 (57.4)	13,729	284	1	0.001	1	0.09
	Lesotho	4,736 (27.2)	6,471	190	1.40 (1.17–1.68)		1.15 (0.95–1.40)	
	Mozambique	1,777 (10.2)	2,499	40	0.84 (0.60–1.18)		0.79 (0.57–1.11)	
	Other	917 (5.3)	1,327	27	1.06 (0.71–1.58)		0.82 (0.54–1.24)	
Years in workforce ^c^	0–9	4,683 (26.9)	6,391	85	1	<0.001		
	10–19	4,753 (27.3)	6,856	147	1.68 (1.28–2.20)			
	20–29	5,511 (31.7)	7,608	217	2.11 (1.64–2.71)			
	≥30	2,446 (14.1)	3,107	90	2.12 (1.57–2.86)			
Type of work	Surface	1,537 (8.8)	2,165	30	1	0.002	1	0.002
	Underground	15,836 (91.2)	21,762	508	1.71 (1.18–2.48)		1.75 (1.20–2.54)	
Type of housing	Hostel	10,239 (58.7)	14,378	364	1	<0.001	1	0.004
	Other	7,206 (41.3)	9,657	177	0.67 (0.55–0.80)		0.74 (0.61–0.91)	
Previous TB	No	15,427 (88.5)	21,413	430	1	<0.001	1	<0.001
	Yes	2,005 (11.5)	2,605	111	2.08 (1.69–2.57)		1.89 (1.52–2.35)	
Self-reported ART use	No	16,956 (97.3)	23,386	523	1	0.42	1	0.64
	Yes	471 (2.7)	625	17	1.23 (0.76–1.99)		0.89 (0.54–1.46)	
Number of	1–2	4,640 (26.6)	6,513	159	1	0.001	1	<0.001
IPT prescriptions ^d,e^	3–5	1,855 (10.6)	2,557	56	0.86 (0.64–1.17)		0.81 (0.59–1.09)	
	6+	10,951 (62.8)	14,967	326	0.79 (0.65–0.97)		0.63 (0.51–0.77)	

^a^ Adjusted for cluster using a fixed effect

^b^ *P* value for departure from linearity 0.002

^c^ *P* value for departure from linearity 0.02

^d^ *P* value for departure from linearity 0.83

^e^ *P* value for linear trend 0.02

^f^ On 17,332 participants, adjusted for all variables shown

aHR, Adjusted hazard ratio; ART, Antiretroviral therapy; CI, Confidence interval; HR, Hazard ratio; IPT, Isoniazid preventive therapy; pyrs, Person-years; TB, Tuberculosis

The risk factor analysis in the AT cohort comprised 16,962 pyrs in the first year after IPT and 10,822 pyrs from the second year onwards. The risk factor estimates were very similar to the ITT analysis (Additional file 1: Table S3).

### Effect modification of risk factors by follow-up period

No evidence for effect modification of risk factors by follow-up period was found; associated risk factors were similar in the first year of follow-up compared to afterwards (Table 3). This included the effect of number of doses of IPT by follow-up time period. The results of this analysis in the AT cohort were very similar (Additional file 1: Table S4).

**Table 3:** Risk factors stratified by time after intended end of isoniazid preventive therapy (intention-to-treat)

		Year 0–1			Year 1+			
Total		pyrs	TB	aHR (95 % CI) ^a^	pyrs	TB	aHR (95 % CI) ^a^	*P* value ^b^
Sex	Male	15,759	344	1	7,277	179	1	0.15
	Female	709	15	0.82 (0.49–1.38)	290	3	0.35 (0.11–1.11)	
Age, years	≤29	2,229	18	1	992	12	1	0.54
	30–39	4,465	81	2.27 (1.36–3.79)	2,196	50	1.90 (1.01–3.56)	
	40–49	6,995	189	3.27 (2.01–5.30)	3,295	87	2.14 (1.17–3.91)	
	≥50	2,779	71	3.01 (1.79–5.06)	1,085	33	2.42 (1.25–4.70)	
Years in workforce	0–9	4,436	58	1	1,956	27	1	0.15
	10–19	4,543	84	1.48 (1.06–2.08)	2,314	63	2.05 (1.30–3.22)	
	20–29	5,213	155	2.23 (1.65–3.02)	2,395	62	1.86 (1.18–2.92)	
	≥30	2,229	61	2.03 (1.41–2.91)	878	29	2.35 (1.39–3.97)	
Type of work	Surface	1,465	20	1	700	10	1	0.98
	Underground	14,932	339	1.71 (1.09–2.69)	6,830	169	1.72 (0.91–3.27)	
Type of housing	Hostel	9,681	236	1	4,697	128	1	0.57
	Other	6,787	123	0.69 (0.55–0.86)	2,870	54	0.62 (0.45–0.85)	
Previous TB	No	14,618	281	1	6,795	149	1	0.63
	Yes	1,838	78	2.15 (1.68–2.77)	767	33	1.93 (1.32–2.81)	
Self-reported ART use	No	16,013	346	1	7,373	177	1	0.82
	Yes	439	12	1.28 (0.72–2.27)	186	5	1.13 (0.46–2.75)	
Number of IPT prescriptions	1–2	4,321	99	1	2,192	60	1	0.86
	3–5	1,715	36	0.88 (0.60–1.29)	842	20	0.84 (0.50–1.39)	
	6+	10,433	224	0.82 (0.65–1.05)	4,534	102	0.79 (0.42–1.46)	

^a^ Adjusted for cluster. ^b^ Test for interaction for factor with time

aHR, Adjusted hazard ratio; ART, Antiretroviral therapy; CI, Confidence interval; HR, Hazard ratio; IPT, Isoniazid preventive therapy; pyrs, Person-years; TB, Tuberculosis

### Comparison between the observed and estimated TB incidence rate after IPT

The observed TB incidence rate during the first year after IPT (ITT analysis) was 2.18/100 pyrs (95 % CI, 1.79–2.66). The crude average incidence rate of TB disease due to reinfection that might have occurred in the first year after IPT was estimated at 1.28 per 100 pyrs. At a cluster level, the estimated rates were consistently lower than the rates observed in the data (Additional file 1: Table S6).

## Discussion

This study, one of only few to specifically address the durability of IPT, analysed the largest study cohort of people on IPT in the ART era. The large number of endpoints allowed for more power than previous studies to examine the timing and risk factors of incident TB after IPT.

TB incidence increased soon after stopping IPT; within 6 months, the TB incidence rate had more than doubled and, within a year, it was within the range of the community rate in the control clusters of the study (2.95 per 100 pyrs; 95 % CI, 2.47–3.52) [6]. The previously published estimated TB incidence rate after IPT in a subset of our study participants was in the same order of magnitude [6]. The trend in incidence rates over time was also comparable, although our much larger sample size allowed us to look at this in more detail by assessing follow-up time in shorter periods. Increasing rates after IPT discontinuation have also been reported in high-prevalence settings in the pre-ART [24, 25] and ART era [4, 9]. In a medium-incidence setting (Brazil), TB incidence rates among TST-positive HIV-positive persons remained low for a median of 4.8 years following the start of IPT [26, 27]. In addition to a lower force of infection, a higher likelihood of cure due to a lower bacillary burden in latent infection was proposed as an explanation for the absence of rebound in their setting compared to in high-prevalence settings.

Our study design does not allow conclusions about the underlying mechanism of TB after IPT. Reinfection as a mechanism for TB after IPT is very likely in this population with a high annual risk of *M. tuberculosis* infection (estimated at 20 % [22]). TB contact studies in resource-limited settings report the highest incidence of TB disease in the first year after acquisition [28], and therefore reinfection could certainly be responsible for some of the TB incidence in the first year after IPT. However, the rapid increase in incidence after stopping seems suggestive of a role for reactivation disease. Mathematical modelling of the rebound in TB incidence in three sub-Saharan African IPT trials in the pre-ART era estimated reactivation of persistent latent infection to be responsible for almost all TB after the end of IPT [29]. A recently published mathematical model of the Thibela study data also found that uncured latent infection among HIV-positive miners may partly explain the lack of population level impact [22].

Our crude estimate of the average incidence rate of TB disease in the first year after IPT that might be attributable due to reinfection [22] was lower than our observed estimate of the TB incidence rate in the first year after IPT. Despite the substantial variation in annual risk of infection, this was consistent across clusters. This supports our hypothesis that a proportion of the post-IPT TB cases could be due to reactivation disease.

The rapid return of the TB incidence rate to baseline levels followed by a stable rate over time could therefore reflect a composite of both mechanisms: reactivation disease gradually replaced by reinfection disease, eventually reaching a steady state of a mixture of the two mechanisms. A similar time pattern in the mechanisms underlying recurrent TB disease after TB treatment was found in a molecular epidemiological study in South Africa, where early recurrences were primarily relapses and later recurrences were primarily reinfection [21]. Further, the episode start date was defined as the treatment start date, which is likely substantially later than the actual episode start date, and could have affected our estimate of the possible contribution of reinfection.

Across southern Africa a high proportion of recurrent TB disease is due to reinfection [18, 21, 30–32]. It has been argued that latent infection confers some protection against reinfection in high-prevalence settings [33]. It is unclear whether this protection could be removed by IPT, rendering the treated population at higher risk of reinfection. In our IPT cohort the TB incidence rate did not exceed that in the control arm, suggesting no increased risk of TB after IPT in our cohort.

Our risk factor analysis showed that a longer duration of IPT led to lower rates of TB after IPT. There was no evidence for waning of this effect over time (≥12 months compared to <12 months), although our analysis may have had too short a follow-up period or may have been underpowered to demonstrate this. An extended duration of IPT further reduced TB incidence among TST- and HIV-positive persons in high incidence settings [7]. However, this effect also waned over time after the end of IPT [9], suggesting that a longer duration of IPT does not increase the durability of protection in high-prevalence settings. Mathematical modelling corroborates this and further estimates that TB disease among those treated with rifamycin-containing preventive therapy is less likely to be due to reactivation, based on a Ugandan study showing a slower rebound in participants having used a regimen including rifamycins compared to only isoniazid [25, 29]. Rifamycins have a higher sterilizing potential; research into the use of regimens which include them is ongoing. Thus far, trials comparing regimens with isoniazid and rifamycin have not shown lower rates of TB in a high transmission setting, although most were not designed to show superiority [34, 35].

The risk factors for TB after IPT identified herein are well-known and the same as before IPT [36], suggesting IPT does not modify these. We found no evidence for different risk factors in the first year compared to later years after IPT. Follow-up might not have been long enough or our analysis might have been underpowered to identify a difference, however. The risk factors we identified mediate risks of reinfection as well as reactivation, thereby not helping to differentiate between the two underlying disease mechanisms.

The strengths of our analysis include the large number of TB diagnoses and person-years of follow-up in a well-characterised employment cohort resulting in higher power than other studies to date to test hypotheses, in particular relating to effect modification. Several important risk factors were not measured and could therefore not be adjusted for, most importantly silicosis and HIV status, which combine multiplicatively [36]. It would have been interesting to stratify the trajectory of TB rates after IPT by HIV status, as HIV-positive patients (especially those with a low CD4 count) might progress to disease faster after reinfection than HIV-negative patients. As the HIV prevalence among the gold miners is higher than among the general population, this might limit the direct comparability of our findings [37]. However, we applied the same HIV prevalence to arrive at our modelled estimate of TB incidence after reinfection, thereby not affecting our conclusions of the contribution of the underlying mechanism of disease.

We were not able to include ART use as a time-updated variable, whereas we know that ART use increased during the course of the study [22] and is highly associated with decreasing risk of TB disease [22, 38]. Our analysis only included self-reported ART usage, which likely represents misclassification; however, removing this variable from the multivariable analysis did not change the estimates. Selection bias may have arisen by a proportion of participants at higher risk of TB leaving the workforce (during or following IPT). Follow-up practices differed during IPT and after IPT, possibly causing ascertainment bias leading to an underestimate of TB incidence after IPT. The analyses did not take account of possible incomplete adherence to IPT, possibly leading to an overestimate of IPT duration and therefore of TB incidence rates during IPT. This potential misclassification would only occur in the AT analysis, thereby not impacting our main outcome and conclusions. Finally, we did not have access to cluster-specific estimates of HIV prevalence, case detection and pre-treatment loss to follow-up. This might have led to an over- or underestimate of the cluster-specific TB incidence rates as calculated by our model.

## Conclusions

The durability of protection by IPT was lost within 6–12 months in this population with a high annual risk of *M. tuberculosis* infection and a high HIV prevalence. The observed TB incidence in the first year after IPT was higher than the crude estimate of the TB incidence attributable to reinfection, suggesting that reactivation of persistent latent infection played a role in the rapid return to baseline TB incidence. Further studies of the durability of alternative (rifamycin-containing) TB preventive therapy regimens, alone or in combination with ART, are warranted.

## Additional file

Additional file 1: Table S1. Reported long-term TB incidence rates in IPT trials. **Figure S1.** Thibela TB study schematic of enrolment and follow-up period within a cluster. **Figure S2.** Rates of TB incidence over time during and after IPT. **Table S2.** Baseline characteristics of the participants included in the risk factor analysis. **Table S3.** Comparison of baseline characteristics of all participants and those who left the workforce for other reasons than illness. **Table S4.** Risk factors of TB incidence after IPT (as-treated). **Table S5.** Risk factors by time after IPT discontinuation (as-treated). **Table S6.** Comparison between observed TB incidence and estimated TB incidence attributable to reinfection in the first 12 months after IPT (intention-to-treat). (DOCX 166 kb)
