# Supplementary material for: The timing of tuberculosis after isoniazid preventive therapy among gold miners in South Africa: a prospective cohort study
Source: BMC Med. 2016 Mar 23;14:45. doi: 10.1186/s12916-016-0589-3 (PMC4804575; doi:10.1186/s12916-016-0589-3)
Supplement: Additional file 1: Table S1. — Reported long-term TB incidence rates in IPT trials. Figure S1. Thibela TB study schematic of enrolment and follow-up period within a cluster. Figure S2. Rates of TB incidence over time during and after IPT. Table S2. Baseline characteristics of the participants included in the risk factor analysis. Table S3. Comparison of baseline characteristics of all participants and those who left the workforce for other reasons than illness. Table S4. Risk factors of TB incidence after IPT (as-treated). Table S5. Risk factors by time after IPT discontinuation (as-treated). Table S6. Comparison between observed TB incidence and estimated TB incidence attributable to reinfection in the first 12 months after IPT (intention-to-treat). (DOCX 166 kb) [file 12916_2016_589_MOESM1_ESM.docx]

# Online supplement

to manuscript

**Tuberculosis after isoniazid preventive therapy among gold miners in South Africa: a prospective cohort study.**

Sabine M. Hermans, Alison D. Grant, Violet Chihota, James J Lewis, Emilia Vynnycky, Gavin J. Churchyard, Katherine L. Fielding

## Table S1. Reported long-term TB incidence rates in IPT trials.

| **First author** | **Years of study** | **Country** | **Background rate^1^** | **Population** | **mean FU (years)** | **N** | **Arm** | **Overall^1,2^** | **Over time^1,2^** |
| --- | --- | --- | --- | --- | --- | --- | --- | --- | --- |
| Horwitz [[1](#_ENREF_1)] | 1956-1962 | Greenland | 1.2-2% | community-wide | NR (max 6) | 3907 | placebo | 1.4 | yr0-1 1.9, yr1-2 1.7, yr2-3 1.4, yr3-4 1.2, yr4-5 1.3, yr5-6 0.8 |
|  |  |  |  |  | NR (max 6) | 4174 | 2x3H (LD) | **1** | **yr0-1 1.3**, yr1-2 1.0, yr2-3 1.0, yr3-4 1.1, yr4-5 0.8, yr5-6 0.6 |
| Ferebee [[2](#_ENREF_2)] | 1957-1960 | United States | NR | mental institutions | NR (max 10) | 12326 | placebo | NR | yr0-1: 0.17, yr2-3: 0.16, yr4-5: 0.07, yr6-7: 0.09, yr8-9: 0.04, yr10: 0.03 |
|  |  |  |  |  | NR (max 10) | 12884 | min 9H | NR | **yr0-1: 0.02**, yr2-3: 0.08, yr4-5: 0.02, yr6-7: 0.06, yr8-9: 0.01, yr10: 0.01 |
| IUAT [[3](#_ENREF_3)] | NR | Eastern Europe^3^ | NR | adults, fibrotic lesions, TST+ | NR (max 5) | 6990 | placebo | NR | yr0-1 4.4, yr1-2 2.6, yr2-3 3.1, yr3-4 2.4, yr4-5 1.6 |
|  |  |  |  |  | NR (max 5) | 6956 | 3H | NR | **yr0-1 2.0**, yr1-2 2.3, yr2-3 2.3, yr3-4 2.1, yr4-5 2.5 |
|  |  |  |  |  | NR (max 5) | 6965 | 6H | NR | **yr0-1 0.7**, yr1-2 0.9, yr2-3 1.0, yr3-4 1.2, yr4-5 1.1 |
|  |  |  |  |  | NR (max 5) | 6919 | 12H | NR | **yr0-1 1.0**, yr1-2 0.8, yr2-3 0.3, yr3-4 1.0, yr4-5 0.0 |
| Comstock [[4](#_ENREF_4)] | 1957-1977 | Alaska | 2% | community-wide | NR (max 13) | 2418 | placebo | 2.8 | NR |
|  |  |  |  |  | NR (max 13) | 2521 | 12H | **1.3** | NR |
| Pape [[5](#_ENREF_5)] | 1986-1989 | Haiti | NR | HIV+, no ART | NR (max 6) | 60 | placebo | 7.5 | median time to TB 29mo |
|  |  |  |  |  | NR (max 6) | 58 | 12H | **2.2** | median time to TB 38mo |
| Halsey [[6](#_ENREF_6)] | 1990-1992 | Haiti | NR | HIV+, TST+, no ART | NR (overall 2.5) | 370 | 6H | **1.7** | NR |
|  |  |  |  |  | NR (overall 2.5) | 380 | 2RP | **1.8** | NR |
| Gordin [[7](#_ENREF_7)] | 1991-1997 | US, Mexico, Haiti, Brazil | NR | HIV+, TST+, no ART | 3.1 | 792 | 12H | **1.2** | NR |
|  |  |  |  |  | 3.1 | 791 | 2RP | **1.2** | NR |
| Quigley^4^ [[8](#_ENREF_8)] | 1992-1994 | Zambia | 5-8% | HIV+, no ART | 2.1 (0-6.9) | 350 | placebo | 4.3 | yr0-1.5: 4, yr1.5-3: 6.1, yr3+: 3.6 |
|  |  |  |  |  | 2.2 (0-7.1) | 352 | 6H | **3** | **yr0-1.5: 1.3**, yr1.5-3: 4.7, yr3+: 3.9 |
|  |  |  |  |  | 2.1 (0-6.9) | 351 | 3RZ | **3.7** | **yr0-1.5: 1.9**, yr1.5-3: 4.4, yr3+: 5.1 |
| Johnson [[9](#_ENREF_9)] | 1993-1995 | Uganda | 4.2 | HIV+, no ART | 2.2 | 787 | placebo | 4.1 | yr1 3.4, yr2 4.8, yr3 4.5, yr4 5.1 |
|  |  |  |  |  | 2.1 | 931 | 6H | **3** | **yr1 2.1**, yr2 3.6, yr3 4.0, yr4 4.2 |
|  |  |  |  |  | 2.1 | 556 | 3RH | **1.9** | **yr1 1.1**, yr2 2.8, yr3 1.8, yr4 3.3 |
|  |  |  |  |  | 1.7 | 462 | 3RHZ | **1.9** | **yr1 2.4**, yr2 1.1, yr3 2.4, yr4 NA |
| Hawken [[10](#_ENREF_10)] | 1992-1994 | Kenya | NR | HIV+, no ART | 1.8 (0-3.4) | 342 | placebo | 3.9 | median time to TB 17(5-35) mo |
|  |  |  |  |  | 1.8 (0-3.4) | 342 | 6H | **4.3** | **median time to TB 14(4-38) mo** |
| Sterling [[11](#_ENREF_11)] | 2001-2008 | US, Canada, Brazil, Spain | 2.5% | adult at risk populations^5^ | 2.6 | 3745 | 6H | **0.2** | 24mo: 0.2 |
|  |  |  |  |  | 2.6 | 3986 | 3RH | **0.1** | 24mo: 0.1 |
| Martinson [[12](#_ENREF_12)] | 2002-2011 | South Africa | 5-10% | HIV+, TST+, no ART | 4 | 328 | 12wRifapH | **2** | NR |
|  |  |  |  |  | 4.1 | 329 | 12wRH | **2** | NR |
|  |  |  |  |  | 3.9 | 164 | contH | **1.4** | NR ^6^ |
|  |  |  |  |  | 3.9 | 327 | 6H | **1.9** | NR |
| Samandari [[13](#_ENREF_13), [14](#_ENREF_14)] | 2004-2006 | Botswana | 3.30% | HIV+, ART roll-out^7^ | 2.6 | 827 | 6H | NR | **0-3yr 1.3**, 3-6yr 1.1 |
|  |  |  |  |  | 2.5 | 853 | 36H | NR | **0-3yr 0.7**, 3-6yr 0.9 |
| Golub [[15-17](#_ENREF_15)] | 2005-2009 | Brazil | 1.1-1.3 ^8^ | HIV+, TST+, ART roll-out^9^ | 4.6 | 1563 | 6H | 0.57 | **IPT:0**, yr1 1.3, yr2 0.6, yr3 0.3, yr4+ 0.6 |
|  |  |  |  |  | 5.5 | 475 | no 6H | 6.74 | NR |
| Churchyard^10^ [[18](#_ENREF_18)] | 2006-2009 | South Africa | 4% | community-wide; miners | 2.2 | 6263 | control | 2.42 | 0-9mo 2.5, 9-18mo 2.3, >18mo 2.6 |
|  |  |  |  |  | 1.97 | 4646 | 9H | 1.87 | **0-9mo 1.0**, 9-18mo 2.2, >18mo 2.2 |
| Rangaka [[19](#_ENREF_19)] | 2007-2011 | South Africa | 4.5 (1.6-5.6) [[20](#_ENREF_20)] | HIV+, on ART | overall: 2.5 | 667 | control | 3.6 | **yr0-1: 3.9**, yr 1-2: 3.7, >yr 2: 3.0 |
|  |  |  |  |  | overall: 2.5 | 662 | 12H | 2.3 | **yr0-1: 2.0**, yr 1-2: 2.2, >yr 2: 2.7 |
| Temprano [[21](#_ENREF_21)] | 2008-2012 | Ivory Coast | NR | HIV+, ART start, high CD4^11^ | 2.5 | 1030 | 6H | 1.1 | NR |
|  |  |  |  |  | 2.5 | 1026 | no 6H | 2.5 | NR |

^1^ Rate/100,000 person-years; ^2^ Figures in bold are or include period on IPT; ^3^ Czechoslovakia, Finland, German Democratic Republic, Hungary, Poland, Romania, Yugoslavia; rates over time abstracted from Figure 1; ^4^ Initial study results reported in Mwinga et al, AIDS 1998; ^5^ HH contacts (70%), TST conversion (25%), HIV (2%), fibrosis on CXR (2%); ^6^ "rate escalated markedly after discontinuation"; ^7^ 2% were on ART at start IPT, 47% at 36 months; ^8^ from Thrio results paper (rates in control and intervention period); ^9^ 67% were on ART at start IPT; ^10^ Results from a subset of the Thibela TB sutdy participants presented in this paper; ^11^ CD4 count above WHO threshold to start ART, below 800 cells/ul

Note. BL, baseline; cont, continuous; FU, follow-up; H, isoniazid; IPT, isoniazid preventive therapy; LD, low dose; mo, months; N, number; NR, not reported; P, placebo; Rifap, rifapentine; TB, tuberculosis; TST, tuberculin skin test; US, United States; w, weeks; yr, year.

To identify all data from IPT trials on long-term TB incidence rates after IPT discontinuation and on how these rates change over time, we performed a literature search using PubMed and by searching reference lists of identified publications, without restrictions on language or publication date. We used the following search terms: tuberculos*; HIV; AIDS; isoniazid; trial; prevention, and the following MeSH terms: Tuberculosis, Pulmonary/epidemiology, Tuberculosis, Pulmonary/prevention & control; Antitubercular Agents/therapeutic use; Isoniazid/therapeutic use; HIV-1. All were trials among adults and excluded participants with active TB.

## Figure S1. Thibela TB study schematic of enrolment and follow-up period within a cluster. Adapted from [[22](#_ENREF_22)]

^^

^1^Estimated size divided by enrolment capacity (30 patients/day, 5 days/week) = defined enrolment period; irrespective of number of enrollees

^2^New recruits were offered IPT, but follow-up ceased at the end of cluster follow-up (<12 months; very small proportion of enrollees).

IPT, isoniazid preventive therapy; time0 = start of time at risk for this analysis.

## Figure S2. Rates of TB incidence over time during and after IPT assuming a stricter TB case definition.

A: intention to treat analysis (intended duration of IPT), B: as-treated analysis (actual duration of IPT). Rates were adjusted for clustering by cluster.

*P* values for overall association between TB incidence and time period of follow-up: A: <0.001 and B: <0.001.

*P* values for tests for linearity in TB incidence rates over time since IPT cessation: A: 0.014 and B: 0.049.

*P* values for tests for departure from linearity in TB incidence rates over time since IPT cessation: A: 0.667 and B: 0.027.

## Table S2. Baseline characteristics of the participants included in the risk factor analysis.

| **Characteristic (n [col%])^1^** |  | **ITT cohort (n [col%])** | **AT cohort (n [col%])** |
| --- | --- | --- | --- |
| **Total** |  | **17445** | **17969** |
| Gender | Male | 16704 (95.8) | 17216 (95.8) |
| Age (years) | <=29 | 2370 (13.6) | 2437 (13.6) |
|  | 30-39 | 4677 (26.8) | 4829 (26.9) |
|  | 40-49 | 7363 (42.2) | 7552 (42) |
|  | 50+ | 3035 (17.4) | 3150 (17.5) |
| Country of origin | SA | 10008 (57.4) | 10298 (57.3) |
|  | Lesotho | 4736 (27.2) | 4885 (27.2) |
|  | Mozambique | 1777 (10.2) | 1826 (10.2) |
|  | Other | 917 (5.3) | 952 (5.3) |
| Years in workforce | 0-9 | 4683 (26.9) | 4814 (26.9) |
|  | 10-19 | 4753 (27.3) | 4911 (27.4) |
|  | 20-29 | 5511 (31.7) | 5653 (31.6) |
|  | 30+ | 2446 (14.1) | 2535 (14.2) |
| Type of work | Underground | 15836 (91.2) | 16318 (91.2) |
| Type of housing | Hostel | 10239 (58.7) | 10524 (58.6) |
| Previous TB | Yes | 2005 (11.5) | 2099 (11.7) |
| Previous IPT | Yes | 73 (0.4) | 77 (0.4) |
| Self-reported ART use | Yes | 471 (2.7) | 497 (2.8) |

ART, antiretroviral therapy; AT, as-treated; IPT, isoniazid preventive therapy; IQR, interquartile range; ITT, intention to treat; N, number; TB, tuberculosis.

^1^Missing data (n): gender (1), age (43), country of origin (8), years in workforce (53), type of work (73), type of residence (1), previous TB (14), previous IPT (34), self-reported ART use (19).

## Table S3. Comparison of baseline characteristics of all participants and those who left the workforce for other reasons than illness.

| **Characteristic (n [col%])^1^** |  | **Total (n [col%])** | **Left work force (n [col%])** | ***P*-value** |
| --- | --- | --- | --- | --- |
| **Total** |  | **18520** | **1772** |  |
| Gender | Male | 17763 (95.9) | 1738 (98.1) | <0.001 |
| Age (years) | <=29 | 2456 (13.3) | 255 (14.4) | <0.001 |
|  | 30-39 | 4919 (26.6) | 404 (22.8) |  |
|  | 40-49 | 7782 (42) | 493 (27.8) |  |
|  | 50+ | 3362 (18.2) | 620 (35) |  |
| Age (mean [SD]) |  | 41 (9) | 43 (11) | <0.001 |
| Country of origin | SA | 10501 (56.7) | 984 (55.6) | 0.001 |
|  | Lesotho | 5178 (28) | 556 (31.4) |  |
|  | Mozambique | 1854 (10) | 148 (8.4) |  |
|  | Other | 979 (5.3) | 82 (4.6) |  |
| Years in workforce (median [IQR]) |  | 18 (8,26) | 21 (10,31) | <0.001 |
| Years in workforce | 0-9 | 4874 (26.4) | 437 (24.8) | <0.001 |
|  | 10-19 | 5017 (27.2) | 379 (21.5) |  |
|  | 20-29 | 5846 (31.7) | 437 (24.8) |  |
|  | 30+ | 2724 (14.8) | 512 (29) |  |
| Type of work | Underground | 16821 (91.2) | 1604 (90.6) | 0.36 |
| Type of housing | Hostel | 10913 (58.9) | 1007 (56.8) | 0.06 |
| Previous TB | Yes | 2212 (12) | 240 (13.5) | 0.03 |
| Previous IPT | Yes | 79 (0.4) | 5 (0.3) | 0.33 |
| Self-reported ART use | Yes | 521 (2.8) | 41 (2.3) | 0.18 |

## Table S4. Risk factors of TB incidence after IPT (as-treated).

| **Total** |  | **pyrs** | **TB** | **HR (95% CI)**^1^ | ***P*-value** | **aHR (95% CI)**^1,5^ | ***P*-value** |
| --- | --- | --- | --- | --- | --- | --- | --- |
| Gender | Male | 26597 | 619 | 1 | 0.01 | 1 | 0.92 |
|  | Female | 1191 | 19 | 0.58 (0.36-0.91) |  | 0.98 (0.61-1.58) |  |
| Age (years)^2^ | ≤29 | 4079 | 36 | 1 | <0.001 | 1 | <0.001 |
|  | 30-39 | 7893 | 159 | 2.32 (1.61-3.33) |  | 2.29 (1.59-3.30) |  |
|  | 40-49 | 11536 | 321 | 3.11 (2.20-4.40) |  | 2.98 (2.09-4.24) |  |
|  | 50+ | 4280 | 122 | 3.13 (2.16-4.56) |  | 2.92 (1.98-4.29) |  |
| Country of | South Africa | 16033 | 330 | 1 | <0.001 | 1 | 0.16 |
| origin | Lesotho | 7338 | 223 | 1.45 (1.22-1.72) |  | 1.18 (0.98-1.41) |  |
|  | Mozambique | 2904 | 53 | 0.97 (0.73-1.31) |  | 0.91 (0.68-1.23) |  |
|  | Other | 1503 | 32 | 1.14 (0.79-1.65) |  | 0.87 (0.60-1.28) |  |
| Years in | 0-9 | 7825 | 100 | 1 | <0.001 |  |  |
| workforce^3^ | 10-19 | 7959 | 176 | 1.82 (1.42-2.33) |  |  |  |
|  | 20-29 | 8481 | 253 | 2.31 (1.83-2.92) |  |  |  |
|  | 30+ | 3437 | 106 | 2.36 (1.79-3.11) |  |  |  |
| Type of work | Surface | 2511 | 34 | 1 | <0.001 | 1 | <0.001 |
|  | Underground | 25155 | 601 | 1.78 (1.26-2.52) |  | 1.80 (1.26-2.55) |  |
| Type of housing | Hostel | 16392 | 423 | 1 | <0.001 | 1 | 0.003 |
|  | Other | 11396 | 215 | 0.66 (0.56-0.79) |  | 0.76 (0.63-0.91) |  |
| Previous TB | No | 24828 | 498 | 1 | <0.001 | 1 | <0.001 |
|  | Yes | 2939 | 140 | 2.34 (1.94-2.83) |  | 2.05 (1.68-2.50) |  |
| Self-reported ART use | No | 27070 | 611 | 1 | 0.03 | 1 | 0.59 |
|  | Yes | 689 | 25 | 1.63 (1.09-2.44) |  | 1.12 (0.74-1.69) |  |
| Number of IPT | 1-2 | 9517 | 228 | 1 | 0.001 | 1 | <0.001 |
| prescriptions^4^ | 3-5 | 3260 | 78 | 0.96 (0.74-1.25) |  | 0.89 (0.69-1.16) |  |
|  | 6+ | 15013 | 332 | 0.83 (0.69-1.00) |  | 0.66 (0.54-0.79) |  |

^1^Adjusted for cluster. *P*-value for departure from linearity ^2^0.002, ^3^<0.001 and ^4^0.65.

^5^on 17852 individuals

aHR, adjusted hazard ratio; CI, confidence interval; HR, hazard ratio; pyrs, person-years; TB, tuberculosis.

## Table S5. Risk factors by time after IPT discontinuation (as-treated).

|  |  | **Year 0-1** |  |  | **Year ≥1** |  |  |  |
| --- | --- | --- | --- | --- | --- | --- | --- | --- |
| **Total** |  | **pyrs** | **TB** | **HR (95% CI)**^1^ | **pyrs** | **TB** | **HR (95% CI)**^1^ | ***P* value**^2^ |
| Gender | Male | 16238 | 375 | 1 | 10358 | 244 | 1 | 0.24 |
|  | Female | 728 | 14 | 0.70 (0.41-1.19) | 464 | 5 | 0.39 (0.16-0.94) |  |
| Age (years) | ≤29 | 2330 | 17 | 1 | 1749 | 19 | 1 | 0.67 |
|  | 30-39 | 4601 | 90 | 2.73 (1.62-4.58) | 3292 | 69 | 1.95 (1.17-3.24) |  |
|  | 40-49 | 7159 | 202 | 3.78 (2.30-6.21) | 4377 | 119 | 2.50 (1.54-4.06) |  |
|  | 50+ | 2876 | 80 | 3.63 (2.14-6.13) | 1404 | 42 | 2.74 (1.59-4.72) |  |
| Country of origin | SA | 9765 | 198 | 1 | 6268 | 132 | 1 | 0.89 |
|  | Lesotho | 4558 | 143 | 1.51 (1.22-1.87) | 2780 | 80 | 1.35 (1.02-1.79) |  |
|  | Mozambique | 1737 | 30 | 0.94 (0.64-1.39) | 1167 | 23 | 1.01 (0.65-1.58) |  |
|  | Other | 901 | 18 | 1.11 (0.68-1.80) | 602 | 14 | 1.19 (0.69-2.07) |  |
| Years in workforce | 0-9 | 4597 | 57 | 1 | 3227 | 43 | 1 | 0.57 |
|  | 10-19 | 4667 | 94 | 1.72 (1.23-2.40) | 3292 | 82 | 1.94 (1.34-2.81) |  |
|  | 20-29 | 5346 | 166 | 2.45 (1.81-3.32) | 3135 | 87 | 2.09 (1.45-3.01) |  |
|  | 30+ | 2305 | 70 | 2.36 (1.66-3.36) | 1133 | 36 | 2.38 (1.52-3.71) |  |
| Type of work | Surface | 1503 | 20 | 1 | 1008 | 14 | 1 | 0.85 |
|  | Underground | 15392 | 369 | 1.83 (1.16-2.87) | 9764 | 232 | 1.71 (0.99-2.93) |  |
| Type of housing | Hostel | 9881 | 193 | 1 | 6443 | 171 | 1 | 0.32 |
|  | Other | 537 | 11 | 0.98 (0.53-1.81) | 376 | 5 | 0.45 (0.19-1.10) |  |
| Previous TB | No | 15042 | 299 | 1 | 9786 | 199 | 1 | 0.88 |
|  | Yes | 1911 | 90 | 2.32 (1.83-2.94) | 1028 | 50 | 2.39 (1.75-3.26) |  |
| Self-reported ART use | No | 16494 | 369 | 1 | 10577 | 242 | 1 | 0.50 |
|  | Yes | 456 | 18 | 1.79 (1.12-2.88) | 234 | 7 | 1.79 (1.12-2.88) |  |
| Number of IPT prescriptions | 1-2 | 4696 | 113 | 1 | 4821 | 115 | 1 | 0.28 |
|  | 3-5 | 1824 | 49 | 1.05 (0.75-1.47) | 1436 | 29 | 0.84 (0.56-1.27) |  |
|  | 6+ | 10447 | 227 | 0.78 (0.62-0.98) | 4566 | 105 | 0.62 (0.37-1.05) |  |

^1^Adjusted for cluster. ^2^Test for interaction; aHR, adjusted hazard ratio; ART, antiretroviral therapy; CI, confidence interval; HR, hazard ratio; IPT, isoniazid preventive therapy; pyrs, person-years; TB, tuberculosis.

## Table S6. Comparison between estimated TB incidence attributable to reinfection and observed TB incidence in the first 12 months after IPT (intention to treat).

| **Cluster** | **ARI*** | **Estimated TB incidence after reinfection (/100pyrs)** | **Observed TB incidence (/100pyrs [95% CI])** |
| --- | --- | --- | --- |
| 1 | 0.11 | 0.83 | 2.12 (1.45-3.09) |
| 2 | 0.17 | 1.30 | 2.15 (1.65-2.79) |
| 3 | 0.16 | 1.22 | 1.74 (1.23-2.46) |
| 4 | 0.10 | 0.71 | 2.20 (1.66-2.91) |
| 5 | 0.20 | 1.77 | 2.93 (2.36-3.63) |
| 6 | 0.18 | 1.47 | 1.31 (0.84-2.06) |
| 7 | 0.21 | 1.91 | 3.15 (2.39-4.15) |
| 8 | 0.12 | 1.00 | 1.58 (1.17-2.15) |
| Total |  | 1.28 | 2.18 (1.79-2.66) |

* Assumed in and plausible values for the average ARI in the mines [[23](#_ENREF_23)].

ARI, annual risk of infection; CI, confidence interval; IPT, isoniazid preventive therapy; pyrs, person-years.

# References

1. Horwitz O, Payne PG, Wilbek E. Epidemiological basis of tuberculosis eradication. 4. The isoniazid trial in Greenland. Bulletin of the World Health Organization. 1966;35(4):509-26.

2. Ferebee SH. Controlled chemoprophylaxis trials in tuberculosis. A general review. Bibl Tuberc. 1970;26:28-106.

3. Efficacy of various durations of isoniazid preventive therapy for tuberculosis: five years of follow-up in the IUAT trial. International Union Against Tuberculosis Committee on Prophylaxis. Bull World Health Organ. 1982;60(4):555-64.

4. Comstock GW, Baum C, Snider DE, Jr. Isoniazid prophylaxis among Alaskan Eskimos: a final report of the bethel isoniazid studies. American Review of Respiratory Disease. 1979;119(5):827-30.

5. Pape JW, Jean SS, Ho JL, Hafner A, Johnson WD, Jr. Effect of isoniazid prophylaxis on incidence of active tuberculosis and progression of HIV infection. Lancet. 1993;342(8866):268-72.

6. Halsey NA, Coberly JS, Desormeaux J, Losikoff P, Atkinson J, Moulton LH et al. Randomised trial of isoniazid versus rifampicin and pyrazinamide for prevention of tuberculosis in HIV-1 infection. Lancet. 1998;351(9105):786-92.

7. Gordin F, Chaisson RE, Matts JP, Miller C, de Lourdes Garcia M, Hafner R et al. Rifampin and pyrazinamide vs isoniazid for prevention of tuberculosis in HIV-infected persons: an international randomized trial. Terry Beirn Community Programs for Clinical Research on AIDS, the Adult AIDS Clinical Trials Group, the Pan American Health Organization, and the Centers for Disease Control and Prevention Study Group. JAMA. 2000;283(11):1445-50.

8. Quigley MA, Mwinga A, Hosp M, Lisse I, Fuchs D, Porter JDH et al. Long-term effect of preventive therapy for tuberculosis in a cohort of HIV-infected Zambian adults. AIDS. 2001;15(2):215-22.

9. Johnson JL, Okwera A, Hom DL, Mayanja H, Mutuluuza Kityo C, Nsubuga P et al. Duration of efficacy of treatment of latent tuberculosis infection in HIV-infected adults. AIDS. 2001;15(16):2137-47.

10. Hawken MP, Meme HK, Elliott LC, Chakaya JM, Morris JS, Githui WA et al. Isoniazid preventive therapy for tuberculosis in HIV-1-infected adults: results of a randomized controlled trial. AIDS. 1997;11(7):875-82.

11. Sterling TR, Villarino ME, Borisov AS, Shang N, Gordin F, Bliven-Sizemore E et al. Three months of rifapentine and isoniazid for latent tuberculosis infection. New England Journal of Medicine. 2011;365(23):2155-66.

12. Martinson NA, Barnes GL, Moulton LH, Msandiwa R, Hausler H, Ram M et al. New regimens to prevent tuberculosis in adults with HIV infection. New England Journal of Medicine. 2011;365(1):11-20.

13. Samandari T, Agizew TB, Nyirenda S, Tedla Z, Sibanda T, Shang N et al. 6-month versus 36-month isoniazid preventive treatment for tuberculosis in adults with HIV infection in Botswana: a randomised, double-blind, placebo-controlled trial. Lancet. 2011;377(9777):1588-98.

14. Samandari T, Agizew TB, Nyirenda S, Tedla Z, Sibanda T, Mosimaneotsile B et al. Tuberculosis incidence after 36 months' isoniazid prophylaxis in HIV-infected adults in Botswana: a posttrial observational analysis. AIDS. 2015;29(3):351-9.

15. Durovni B, Cavalcante SC, Saraceni V, Vellozo V, Israel G, King BS et al. The implementation of isoniazid preventive therapy in HIV clinics: the experience from the TB/HIV in Rio (THRio) study. AIDS. 2010;24 Suppl 5:S49-56.

16. Golub JE, Saraceni V, Cavalcante SC, Cohn S, Pacheco AG, Lau B et al. Durability of isoniazid preventive therapy in HIV-positive patients in a medium burden TB setting. 20th Conference on Retroviruses and Opportunistic Infections; Atlanta, GA, USA2012. p. Poster 190 LB.

17. Golub JE, Cohn S, Saraceni V, Cavalcante SC, Pacheco AG, Moulton LH et al. Long-term Protection From Isoniazid Preventive Therapy for Tuberculosis in HIV-Infected Patients in a Medium-Burden Tuberculosis Setting: The TB/HIV in Rio (THRio) Study. Clinical Infectious Diseases. 2015;60(4):639-45.

18. Churchyard GJ, Fielding KL, Lewis JJ, Coetzee L, Corbett EL, Godfrey-Faussett P et al. A trial of mass isoniazid preventive therapy for tuberculosis control. New England Journal of Medicine. 2014;370(4):301-10.

19. Rangaka M, Wilkinson RJ, Boulle A, Glynn JR, Fielding K, Van Cutsem G et al. Isoniazid plus antiretroviral therapy to prevent tuberculosis: a randomised double-blind placebo-controlled trial. Lancet. 2014;384(9944):682-90.

20. Lawn SD, Myer L, Bekker LG, Wood R. Burden of tuberculosis in an antiretroviral treatment programme in sub-Saharan Africa: impact on treatment outcomes and implications for tuberculosis control. AIDS. 2006;20(12):1605-12.

21. A Trial of Early Antiretrovirals and Isoniazid Preventive Therapy in Africa. New England Journal of Medicine. 2015;373(9):808-22.

22. Fielding KL, Grant AD, Hayes RJ, Chaisson RE, Corbett EL, Churchyard GJ. Thibela TB: design and methods of a cluster randomised trial of the effect of community-wide isoniazid preventive therapy on tuberculosis amongst gold miners in South Africa. Contemporary Clinical Trials. 2011;32(3):382-92.

23. Vynnycky E, Sumner T, Fielding KL, Lewis JJ, Cox AP, Hayes RJ et al. Tuberculosis control in South african gold mines: mathematical modeling of a trial of community-wide isoniazid preventive therapy. American Journal of Epidemiology. 2015;181(8):619-32.
